# The impact of the modified schedules of anatomy education on students’ performance and satisfaction: Responding to COVID-19 pandemic in South Korea

**DOI:** 10.1371/journal.pone.0266426

**Published:** 2022-04-11

**Authors:** Young Hyun Yun, Dong Hyun Jo, Su Kyoung Jeon, Hyeok Yi Kwon, Yu Mi Jeon, Dong Hoon Shin, Hyung Jin Choi

**Affiliations:** 1 Department of Anatomy and Cell Biology, Seoul National University College of Medicine, Seoul, South Korea; 2 Department of Biomedical Sciences, Seoul National University College of Medicine, Seoul, South Korea; Karnali Academy of Health Sciences, NEPAL

## Abstract

**Background:**

The coronavirus disease 2019 (COVID-19) pandemic substantially undermined medical education and healthcare systems. Owing to the pandemic in South Korea, most medical schools needed to be flexible when conducting online and offline classes, but the guidelines did not reflect the specificity of medical schools. This study described the impact of modified anatomy education schedules at the Seoul National University College of Medicine (SNUCM) on students’ academic performance and satisfaction.

**Methods:**

Anatomy education in SNUCM is divided into three regional units (the upper and lower limbs, trunk, and head and neck). Owing to the COVID-19 pandemic, the schedule was mixed with simultaneous and rotating schedules. The authors conducted exceptions for online lectures, cadaver dissections, and written and practical examinations in three classes of approximately 50 students each. Furthermore, the authors assessed students’ performance using three sets of written and practical examinations, and students completed a questionnaire regarding modified anatomy laboratory schedules.

**Results:**

Despite the pandemic events in Seoul and South Korea during the laboratory sessions, all sessions were completed without any confirmed COVID-19 cases among the students, faculty, and staff. Most of the scores on the written and practical examinations significantly decreased in 2020 compared to those in 2019. However, in the trunk session that used the virtual anatomy application, the score on the practical examination in 2020 was significantly higher than that in 2019. Over 70% (79 and 77 out of 105 respondents on the upper and lower limbs and trunk, respectively) and 53% (55/105) students reported that there were no significant difficulties in studying anatomy in a face-to-face laboratory.

**Conclusions:**

In conclusion, an adequate education program for cadaver dissection should be developed and provided to overcome the pandemic restrictions. The study findings could serve as a reference for anatomy education during the COVID-19 pandemic.

## Introduction

In late December 2019, coronavirus disease 2019 (COVID-19) first emerged in Wuhan, Hubei Province, China, and spread worldwide [1]. COVID-19 is a new virus belonging to the same family as severe acute respiratory syndrome coronavirus 2 (SARS-coV-2) and some types of common cold [2]. The unprecedented outbreak of COVID-19 has caused great confusion not only in politics, economy, society, and culture, but also in education worldwide, and has become a turning point for introducing a new way of education [3, 4]. Although there have been movements of transformation in medical education, such as the use of online education and virtual reality technology [5], COVID-19 has accelerated this change as students are forced to exercise social distancing [6]. Being highly transmissible, the virus has made it difficult to continue lectures as usual [7].

It is customary that an academic year in a university in South Korea has two semesters: the spring semester from March to June and the fall semester from September to December. First-year medical students began a human anatomy course in March 2020 at the Seoul National University College of Medicine (SNUCM). In March, it was expected that the pandemic would be controlled in several weeks and face-to-face classes would be possible by June. However, the pandemic situation did not improve, and many Korean universities have decided that all lectures should be given online throughout the semester. Due to the temporary closure of medical schools and suspension, medical schools began to convert medical education from traditional forms of face-to-face lecture-based teaching and cadaver dissection to digital formats [8, 9]. Some commonly proposed methods include online learning platforms, mobile applications, and three-dimensional (3D) anatomy models [10]. For medical students, anatomy education is not only important in terms of knowledge and skills for an accurate understanding of human anatomical structures, but it also takes a lot more than dissection and learning [11, 12]. Students can learn the essential traits and virtues of physicians through cadaver dissections. Therefore, we intended to maintain adequate exposure of the students to cadaveric dissections.

The immediate responses to COVID-19 have been documented in several publications, highlighting the innovations and creative instruction pursued by anatomy educators. Several previous studies have highlighted the importance of the potential implications of integrating digital technologies into medical education for the future of learning and assessment [13–15]. In addition, some studies have reported that various technological solutions aid in minimizing the disruption to medical education during the COVID-19 pandemic [16–18]. According to a study conducted by Owolabi and Bekele, adopting multiple learning methods and blended learning might facilitate the ease of adaptation during COVID-19 [19]. This essay provided various materials, including media audio, videos, computer-based educational programs and technology, and simulations. According to another study, students prefer both asynchronous (55%) and synchronous (45%) modes of learning; therefore, a balance between the two modes should be maintained [20]. Yoo et al. assessed students’ achievement between the blended learning group and the traditional learning group and evaluated feedback from students about online lectures in 2021 [21]. Furthermore, several studies on anatomy education have been conducted, including distance education but not face-to-face education [22–24]. While the studies mentioned previously provided insight into the early adaptations to gross anatomy education during COVID-19, they were descriptive in nature and did not include inferential statistics comparing practical examinations and surveys in each session involving the upper and lower limbs, trunk, and head and neck during COVID-19. Furthermore, to the best of our knowledge, there are no publications that provide a detailed example of the modified schedules and methods of teaching anatomy in the early stages of the epidemic, as well as their influence on academic accomplishment and student satisfaction.

The aim of this study was to overcome the limitations of these previously reported studies and to determine whether the modified schedules of anatomy education due to the COVID-19 pandemic affect students’ outcomes and satisfaction in the gross anatomy course. Three specific research questions guided this study: (1) Are there differences in students’ academic achievement between the 2019 and 2020 classes?; (2) What factors contribute to students’ academic achievement?; and (3) What is the level of student satisfaction with the modified schedules of anatomy education?

## Methods

### Subjects

The subjects of the study were first-year medical students who took a human anatomy course at the SNUCM. In 2020, there were 145 students in their first year of medical courses.

### Change in the schedules and methods of teaching for “human anatomy” lecture

In 2020, the total number of class hours (48 hours, divided into three regional units: the upper and lower limbs, trunk, and head and neck) remained the same as in 2019; however, all lectures were replaced with online lectures. The reorganization of the schedules and methods of teaching was influenced by the COVID-19 pandemic in various ways, including through pre-recorded video lectures and online platforms. After two weeks of curriculum suspension due to the COVID-19 pandemic, first-year students were provided with electronic learning (e-learning) materials on gross anatomy from March 20, 2020, to May 1, 2020 (Table 1). E-learning materials, including slides, videos, and lecture notes, were received from professors at least a week before the online posting on e-Teaching and Learning, an e-learning service operated by Seoul National University (SNU). Students can access e-learning materials via the SNUCM electronic learning system. Learning through video lectures lasted for approximately one hour. In accordance with the schedule of cadaver dissection, students were required to attend online lectures before conducting face-to-face cadaver dissection. The table of contents contains a comprehensive listing of human anatomy offered at the SNUCM. A general overview from March 20, 2020, to March 23, 2020, provided approaches for studying anatomy, including regional, systemic, and clinical anatomy. In addition, there were three parts of the examination: the upper and lower limbs, trunk, and head and neck. Written and practical examinations were conducted for 2 days, one day at a time.

**Table 1:** Table of contents and schedule for the human anatomy course according to the restrictions of the COVID-19 pandemic, SNUCM, 2020.

Contents	Deadline for e-learning Materials	Upload date of e-learning Materials	Anatomy Laboratory (face-to-face)
General overview 1	Mar 13	Mar 20	-
General overview 2	Mar 13	Mar 20	-
Introduction to anatomy laboratory	-	-	May 04
General overview 3	Mar 16	Mar 23	-
**Upper/lower limbs**			
Superficial structure of lower limb	Mar 16	Mar 23	May 04
Organization of thigh muscles	Mar 17	Mar 24	May 06
Gluteal region	Mar 19	Mar 26	May 07
Knee and poster thigh muscles			
Leg	Mar 19	Mar 26	May 08
Foot	Mar 20	Mar 27	May 11
Joints of lower limb	Mar 20	Mar 27	
Shoulder	Mar 25	Apr 01	May 12
Axilla	Mar 25	Apr 01	May 13
Thorax	Mar 30	Apr 06	
Arm	Mar 31	Apr 07	May 14
Forearm	Mar 31	Apr 07	
Hand	Apr 01	Apr 08	
Joints of upper limb	Apr 01	Apr 08	
**1**^**st**^ **written and practical examinations**			
**Trunk**			
Thoracic wall and mediastinum	Apr 02	Apr 09	May 18
Pleurae and lung			
Heart	Apr 03	Apr 10	
Fascia, muscles, vessels, and nerves of back	Apr 06	Apr 13	May 19
Anterolateral abdominal wall	Apr 06	Apr 13	May 19
Pelvic cavity, pelvis, and perineum	Apr 07	Apr 14	
Peritoneum and abdominal viscera	Apr 09	Apr 16	May 20
Esophagus, stomach, small intestine, and large intestine	Apr 09	Apr 16	
Liver, pancreas, and spleen	Apr 10	Apr 17	
Posterior abdominal wall	Apr 10	Apr 17	May 20
Pelvic cavity, and perineum	Apr 14	Apr 21	May 21
Vessels, nerves, and lymph of pelvic cavity	Apr 14	Apr 21	
Rectum and anal canal	Apr 15	Apr 22	May 21
Male internal genital organs	Apr 16	Apr 23	May 22
Female internal genital organs	Apr 16	Apr 23	
Medical imaging of abdomen	Apr 15	Apr 22	
**2**^**nd**^ **written and practical examinations**			
**Head/neck**			
Cranium	Apr 17	Apr 24	May 25
Scalp, and cranial meninges	Apr 17	Apr 24	May 26
Face	Apr 17	Apr 24	May 25
Vasculature of brain	Apr 20	Apr 27	May 26
Brain and cranial nerves	Apr 20	Apr 27	
Orbit, and orbital contents	Apr 21	Apr 28	May 27
Eyelids and lacrimal apparatus	Apr 21	Apr 28	
Superficial structures of neck	Apr 22	Apr 29	May 28
Triangles of neck	Apr 22	Apr 29	
Arteries, veins, and nerves in root neck	Apr 22	Apr 29	
Salivary glands, and temporomandibular joint	Apr 22	Apr 29	May 29
Nose, and larynx	Apr 24	May 01	Jun 01
Ear	Apr 24	May 01	
Oral region	Apr 24	May 01	
Medical imaging of head and neck	Apr 24	May 01	
**3**^**rd**^ **written and practical examinations**			

Abbreviation: COVID-19 = coronavirus disease 2019, SNUCM = Seoul National University College of Medicine, e-learning = electronic learning.

### Change in the schedules and methods of teaching for “human anatomy” laboratory sessions

**Fig 1:**
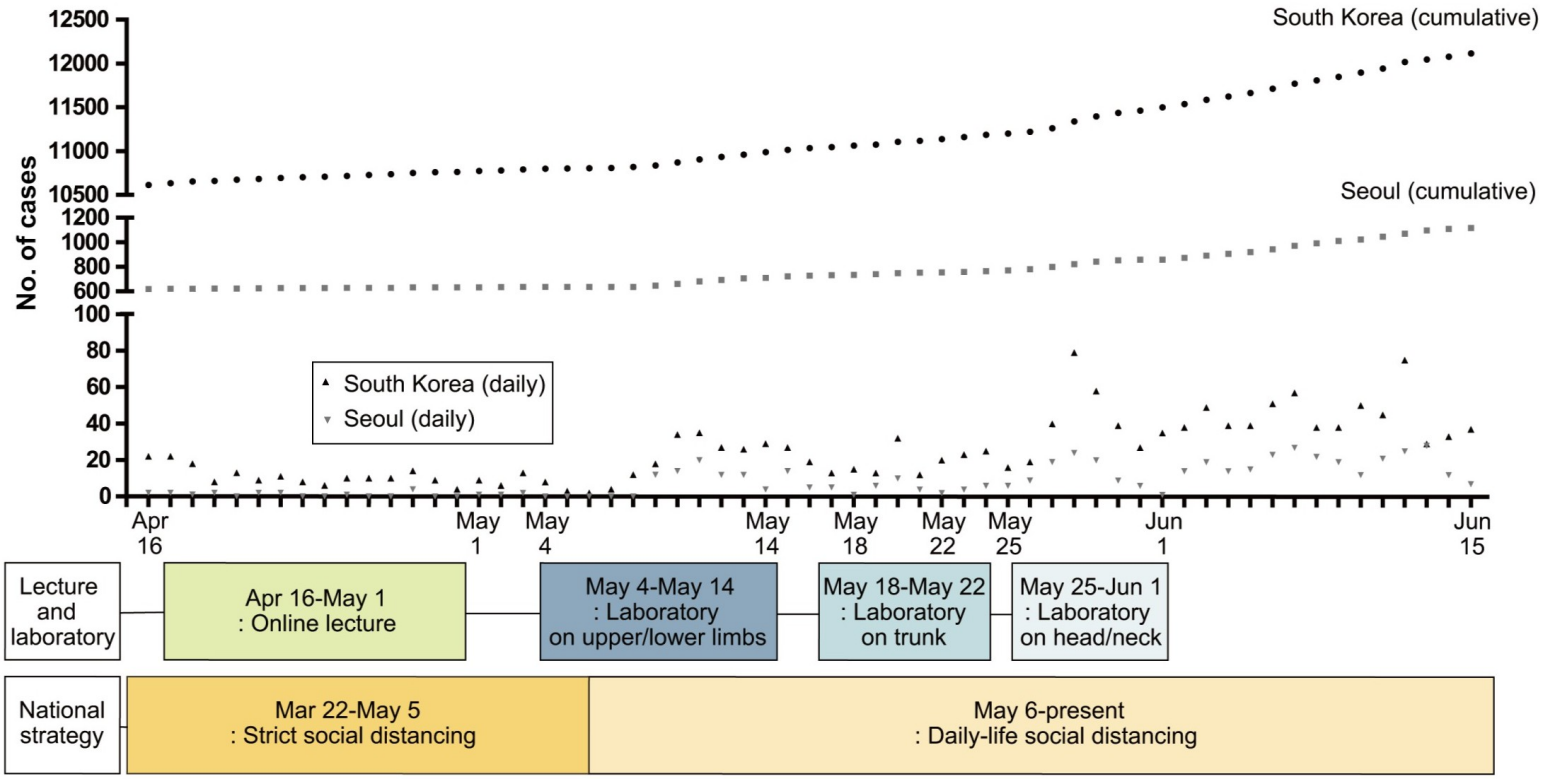
Timeline of cases with COVID-19 (South Korea and Seoul) and schedules of anatomical education in SNUCM. Abbreviation: COVID-19 = coronavirus disease 2019, SNUCM = Seoul National University College of Medicine.

When the government reduced the degree of social distancing in early May 2020, SNUCM prepared face-to-face laboratory sessions for medical students. When new COVID-19 cases in Seoul decreased to zero on May 4 (Fig 1), the face-to-face anatomy laboratory started with simultaneous and rotating schedules [25] until June 1, 2020. There were 24 rotating dissections (3 h each, three classes) and 21 rotating reviews (3 h, three classes). The schedule was modified to fit the number of students in the laboratory facility to approximately 50 per session to increase social distance. The 154 students who participated in the anatomy laboratory were divided into three classes (only one-third of all students participated in a hands-on dissection at a time), and each class conducted a cadaver dissection for 3 h. In a three-hour laboratory dissection session, approximately 50 students were divided into 10 groups, with five students per cadaver. Each group was 3 m from the other groups. The Human Anatomy 7^th^ edition guidelines (Korea Medical Book Publishing Company, Seoul, Korea) were used as the main textbook for cadaver dissection. SNUCM also provides e-Anatomy^®^ (Panmun Education, Seoul, Korea) videos, which provide guidelines for organ-specific fine and overall dissection of human anatomy, and Complete Anatomy^®^ (Elsevier, Amsterdam, Netherlands), an educational 3D anatomy platform.

### Written and practical examinations

Student performance was assessed using three sets of written and practical examinations on three regional units: the upper and lower limbs, trunk, and head and neck. The assessment was conducted mainly by taking a written examination in the morning and a practical examination in the afternoon. The written examination was designed to take place simultaneously in three different classrooms with approximately 50 students at least 2 m apart to avoid contagion. All students had lunch after the written examination. After a lunch break, the students waited at their own written examination sites again and then moved to the practical examination waiting room. It was controlled by teaching assistants so that students who had already taken the practical examination did not meet the next students who came to the waiting area to take the examination. The entrance and exit to the practical examination were separated so that the lines did not overlap with other students. When students left the lab, they threw away the masks they wore during the exam and were provided with new masks and hand sanitizer.

### Survey

An online survey was conducted at the end of the spring 2020 semester. Students were asked to respond using 3- and 5-point Likert-type scale scores regarding the modified anatomy laboratory schedules (S1 Appendix). The questionnaire included the following items: (1) overall satisfaction with the face-to-face anatomy laboratory sessions; (2) preference for the modified anatomy schedules; (3) usage of allotted time for cadaver dissection; (4) preference for 3D digital anatomy educational software; (5) difficulty in learning the human anatomy course; and (6) difficulty in taking practical examinations.

### Statistical analysis

All statistical analyses were performed using SPSS software, version 26 (IBM Crop). We used Student’s t-test to assess the differences in examination scores between 2019 and 2020 and considered a 2-tailed *P* < 0.05 to be statistically significant.

### Ethical consideration

The present study was approved by the Institutional Review Board (IRB) of Seoul National University College of Medicine (IRB number: E-2202-034-1298). It was entirely retrospective (using existing student surveys and grades), and a consent waiver was permitted through the IRB.

## Results

During the anatomy dissection sessions, three students reported symptoms (cough, fever/myalgia, and fever) and were referred for SARS-CoV-2 testing. They were excluded from the laboratory until the results were negative. Despite the ongoing pandemic events in Seoul and South Korea during the laboratory sessions (Fig 1), all sessions were completed without any confirmed cases among the students, faculty members, and staff.

### Academic achievement

**Fig 2:**
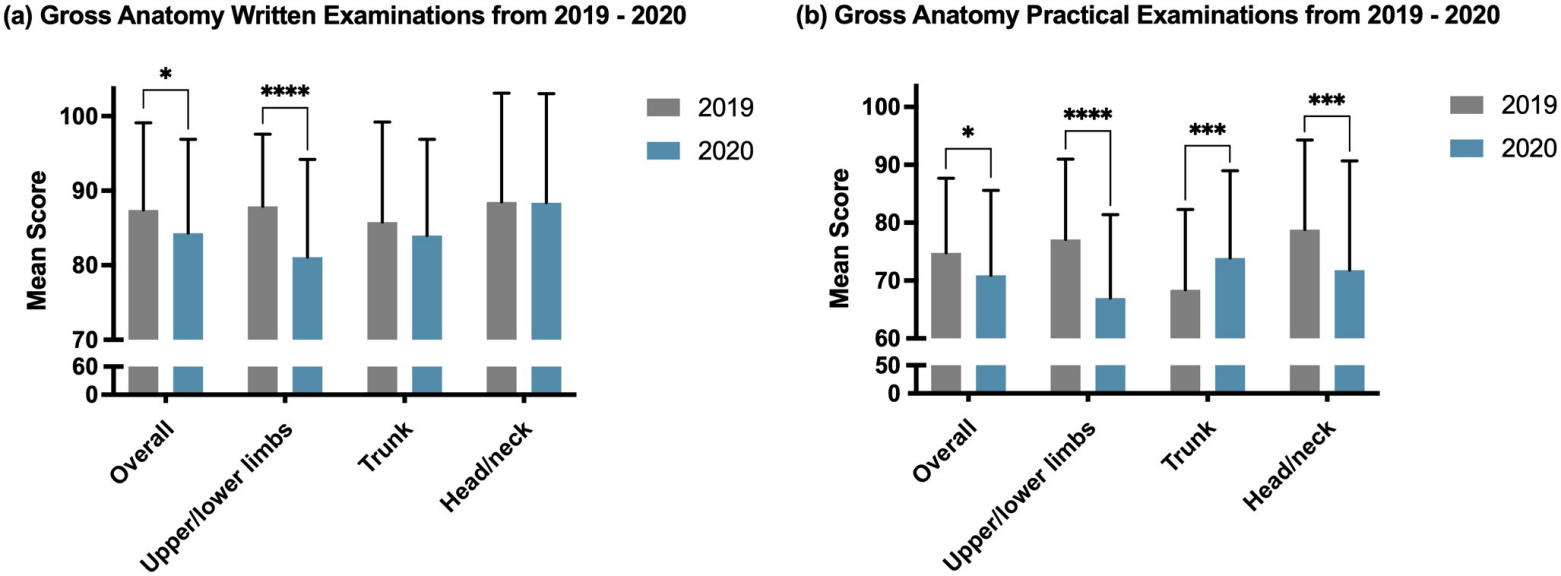
Comparison of written and practical examination scores between 2019 and 2020 at SNUCM. The 2020 schedule of anatomy education was modified because of COVID-19. (a) Students’ performance was assessed using three sets of written examination on three regional units: the upper and lower limbs, trunk, and head and neck. (b) Students’ performance was assessed using three sets of practical examination on three regional units: the upper and lower limbs, trunk, and head and neck. The statistical significance is expressed with the following symbols: * p < 0.05, ** p < 0.01, ***p < 0.001, ****p < 0.0001 to indicate statistical differences. Abbreviation: COVID-19 = coronavirus disease 2019, SNUCM = Seoul National University College of Medicine.

The anatomy assessment scores of the 2019 and 2020 classes, including written and practical examinations, were compared (Fig 2). The total mean scores, including written and practical examinations, were significantly lower in 2020 than in 2019 (p < 0.05). In the written examination in 2020, the upper and lower limb sessions had significantly lower mean scores (p < 0.0001), while there was no difference in the trunk and head and neck sessions (Fig 2a). In the 2020 practical examination, the scores of the upper and lower limbs (p < 0.0001) and head and neck (p < 0.001) sessions were significantly lower than those of 2019 (Fig 2b). In contrast, the score of the trunk session in 2020 was significantly higher than that of the 2019 class (p < 0.01) (Fig 2b).

### Survey results

**Fig 3:**
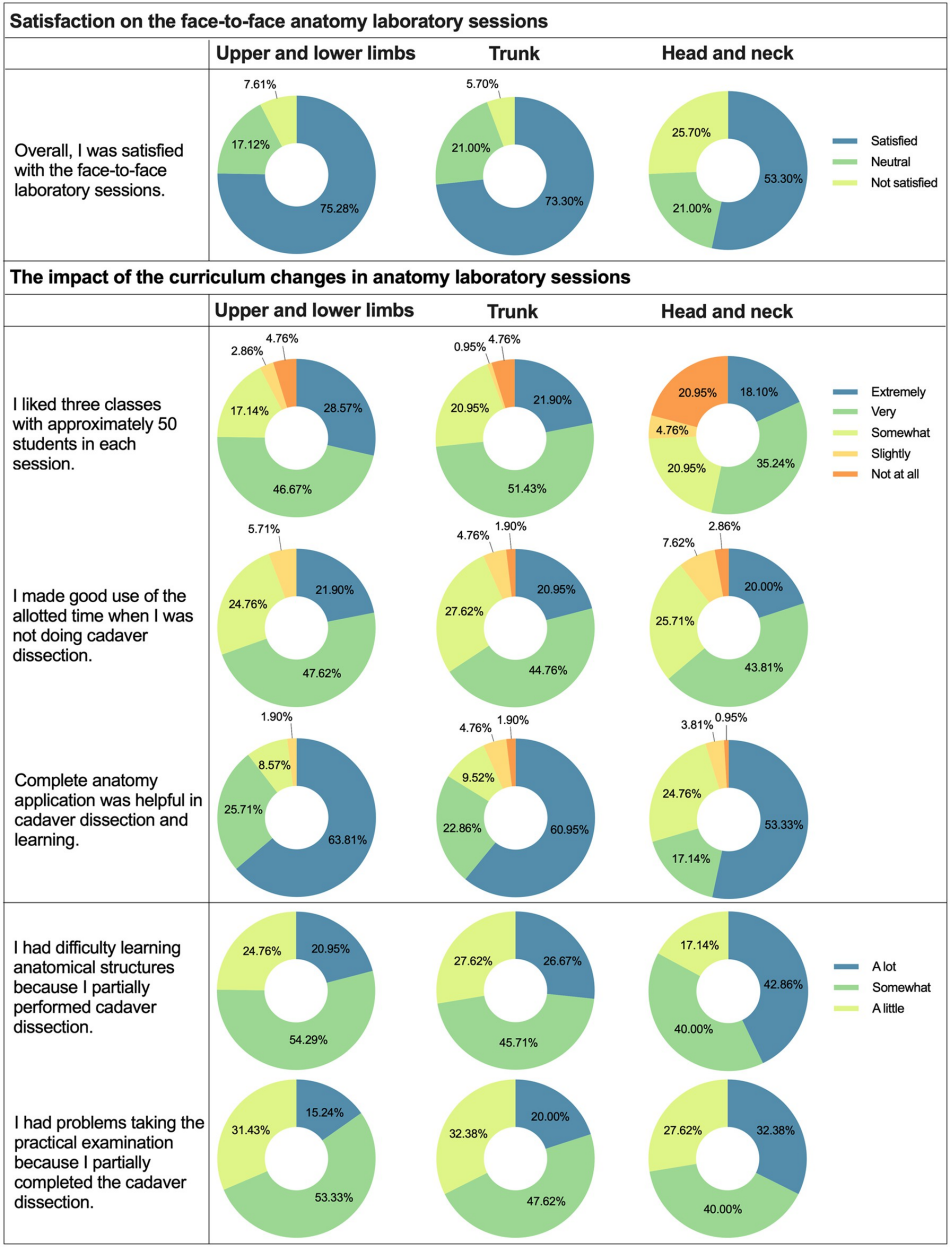
Students’ satisfaction on face-to-face anatomy laboratory sessions. Students’ (n = 105) satisfaction on face-to-face anatomy laboratory sessions was ranked on a 3-point Likert-type scale (1 = satisfied and 3 = not satisfied). Students were asked about the impact of the modified schedules and methods of teaching in anatomy laboratory sessions they experienced after the COVID-19 pandemic, as represented by pie graphs. The 3- and 5-point Likert scale scores are shown in percentage. Abbreviation: COVID-19 = coronavirus disease 2019.

Students (n = 105) who participated in the survey were asked about their satisfaction with the face-to-face anatomy laboratory sessions (Fig 3). Over 50% of the students were satisfied with the upper and lower limbs (75.3%), trunk (77.3%), and head and neck (53.3%) sessions. In addition, the students assessed the impact of the modified schedules and methods of teaching in anatomy laboratory sessions. The modified schedule had several advantages. Most students answered that they preferred three classes with approximately 50 students each in the upper and lower limb (extremely likely: 28.6%, very likely: n = 46.7%), trunk (extremely likely: 21.9%, very likely: 51.4%), and head and neck (extremely likely: 18.1%, very likely: 35.2%) sessions. Moreover, more than half of the students made good use of allotted time for self-directed learning in all sessions (upper and lower limbs: 69.5%, trunk: 65.7%, and head and neck: 63.8%). The largest number of students in the upper and lower limbs (n = 67), trunk (n = 64), and head and neck (n = 56) actively used the 3D atlas Complete Anatomy^®^ and received considerable help. In contrast, among all sessions, the head and neck session was the most difficult for students to learn about anatomical structures (42.9%) and take practical examinations (32.4%).

## Discussion

The purpose of our study was to provide educational schedules and methods for teaching anatomy education in the COVID-19 era and investigate the research outcomes related to students’ academic performance, satisfaction, and factors affecting them. From the perspective of responding to infectious diseases or similar disasters that may occur in the future and preparing for the future of anatomy education, we provide a detailed example of a modified educational schedule and method of teaching.

Regarding anatomy education in the COVID era, this study provided a detailed education schedule and methods of teaching anatomy, which can be applied in the future globally. Although several studies have reported online education in anatomy during the pandemic era [8–10, 13–18], no study has provided a detailed schedule and content allocation. Moreover, the alternate dissection strategy (three teams rotating) could be another alternative for face-to-face anatomy laboratories during the pandemic [25]. In fact, the strategy solved the crowding problem in the dissection laboratory while having consequences for student learning on students’ learning outcomes. Therefore, the face-to-face dissection strategy and detailed educational schedule information of our research can play a complementary role in the COVID-19 era.

Our study results revealed that students’ achievement scores on written and practical examinations in 2020 were significantly lower than those in 2019 (Fig 2). To the best of our knowledge, our results are the first to provide quantitative evidence of the risk of decreased academic performance in anatomical education due to the COVID-19 pandemic, even with efforts such as continuing face-to-face anatomy laboratories. The analysis of our survey revealed that students had difficulty learning anatomical structures and taking practical examinations due to the time limitation of the anatomy laboratory that each student could experience. However, most previous studies on anatomy education have reported that academic achievement was higher or similar to that before the COVID-19 pandemic [21, 26, 27]. These studies suggested positive factors that influence students’ academic achievement but did not provide evidence of the pandemic’s negative impact on academic achievement.

The findings of this study provide unique evidence that adequate supplementation can potentially improve academic achievement in anatomy education. Although overall achievement was lower in 2020 than in 2019, an interesting finding was that the written examination in the trunk and head and neck sessions showed no statistically significant difference in scores between 2020 and 2019 (Fig 2a). Moreover, the practical examination score in the trunk session was significantly higher in 2020 than in 2019 (Fig 2b). All medical students were provided with 3D digital anatomy educational software, but only professors in charge of the trunk session used the software when making lecture materials. In addition, according to the survey, although the opportunity to participate in the laboratory sessions was reduced to only one-half, the software helped in cadaver dissection and learning anatomy (Fig 3).

This study highlights the benefits of integrating digital anatomy educational software with face-to-face anatomy laboratories in medical education. This finding is consistent with previous studies reporting that digital anatomy educational software provides a positive experience [28] and better results on examinations [29]. Therefore, we suggest that the use of digital anatomy educational software helps students quickly understand anatomical structures and their adjacent relationships. As highlighted in this study, the benefits of integrating 3D atlas applications and face-to-face anatomy laboratories in medical education have been demonstrated. Although cadaveric dissection is a useful tool for anatomy education in many medical schools [30], the inevitable change in medical education owing to COVID-19 is still ongoing [5]. Traditional cadaveric dissection cannot be overruled but can be complemented with advanced high-tech innovations, particularly in the pandemic era.

This is the first study to conduct a survey on students’ satisfaction and preference by each session including upper and lower limbs, trunk, and head and neck in anatomy education during the COVID-19 pandemic (Fig 3). Previous studies have investigated students’ satisfaction and preference for learning anatomy in a more comprehensive way and did not analyze the cause of satisfaction [21–24]. One study reported students’ perception of the difficulty associated with dissecting anatomical regions [31]. This study, however, lacked in situational elements, such as class hours, teaching methods, and schedules, implying that students’ perceptions of anatomical regions were limited. In this study, each situational element survey yielded different results. Satisfaction levels differed between the three anatomical regions. Therefore, it is important to conduct separate surveys for each anatomical region.

Several factors contribute to students’ satisfaction. We believe that the continuation of face-to-face cadaver dissection laboratory sessions, even after the COVID-19 pandemic, has contributed to the high level of overall student satisfaction. Furthermore, providing proper personal protective equipment (PPE) and adhering to social distancing guidelines may have contributed to high satisfaction. In addition, the duration of laboratory dissection experience could have been the cause of satisfaction. High satisfaction was observed for upper and lower limbs, and trunk sessions, which had the same duration as in 2019. However, low satisfaction was observed for head and neck sessions, which had a shorter duration than that of 2019. There was no opportunity to preview and review owing to a lack of time, and it would have been difficult to adapt because of a rapid change in schedules.

Our study had several limitations that should be considered. This study had a single-center design at a single institution. Our modified schedules of anatomy education and outcomes may not be applicable to other schools because each medical school has different circumstances. In addition, due to time constraints following the COVID-19 outbreak, it was not possible to validate the instruments used in the present study, and we used a limited number of questionnaire items to measure the level of students’ satisfaction with the human anatomy course. Therefore, further research is required in this regard. Despite these limitations, this study provides supportive evidence for the 2020 anatomy course in response to COVID-19.

In conclusion, in the era of infectious diseases, the quality of education is critically dependent on the planning and preparation of on-off lectures, cadaver dissection, and digital educational software. Our findings provide important insights regarding digital educational software and detailed examples of anatomy education schedules during the COVID-19 pandemic era.

## Supporting information

S1 Appendix A survey on the operation of human anatomy laboratory sessions (the upper and lower limbs, trunk, and head and neck) in 2020. (DOCX)

## References

[1] Harapan H, Itoh N, Yufika A, Winardi W, Keam S, Te H, et al. Coronavirus disease 2019 (COVID-19): A literature review. J Infect Public Health. 2020;13(5):667–73. doi: 10.1016/j.jiph.2020.03.019 32340833 PMC7142680

[2] Pal M, Berhanu G, Desalegn C, Kandi V. Severe Acute Respiratory Syndrome Coronavirus-2 (SARS-CoV-2): An Update. Cureus. 2020;12(3):e7423. doi: 10.7759/cureus.7423 32337143 PMC7182166

[3] Gonzalez T, de la Rubia MA, Hincz KP, Comas-Lopez M, Subirats L, Fort S, et al. Influence of COVID-19 confinement on students’ performance in higher education. PLoS One. 2020;15(10):e0239490. doi: 10.1371/journal.pone.0239490 33035228 PMC7546684

[4] Woolliscroft JO. Innovation in Response to the COVID-19 Pandemic Crisis. Acad Med. 2020;95(8):1140–2. doi: 10.1097/ACM.0000000000003402 32282372 PMC7188042

[5] Emanuel EJ. The Inevitable Reimagining of Medical Education. JAMA. 2020;323(12):1127–8. doi: 10.1001/jama.2020.1227 32105294

[6] Evans DJR, Bay BH, Wilson TD, Smith CF, Lachman N, Pawlina W. Going Virtual to Support Anatomy Education: A STOPGAP in the Midst of the Covid-19 Pandemic. Anat Sci Educ. 2020;13(3):279–83. doi: 10.1002/ase.1963 32277598

[7] Sklar DP. COVID-19: Lessons From the Disaster That Can Improve Health Professions Education. Acad Med. 2020;95(11):1631–3. doi: 10.1097/ACM.0000000000003547 32544103 PMC7309647

[8] Rose S. Medical Student Education in the Time of COVID-19. JAMA. 2020;323(21):2131–2. doi: 10.1001/jama.2020.5227 32232420

[9] Sandhu P, de Wolf M. The impact of COVID-19 on the undergraduate medical curriculum. Med Educ Online. 2020;25(1):1764740. doi: 10.1080/10872981.2020.1764740 32400298 PMC7269089

[10] Dawidziuk A, Kawka M, Szyszka B, Wadunde I, Ghimire A. Global Access to Technology-Enhanced Medical Education During the COVID-19 Pandemic: The Role of Students in Narrowing the Gap. Glob Health Sci Pract. 2021;9(1):10–4. doi: 10.9745/GHSP-D-20-00455 33795360 PMC8087424

[11] Turney BW. Anatomy in a modern medical curriculum. Ann R Coll Surg Engl. 2007;89(2):104–7. doi: 10.1308/003588407X168244 17346399 PMC1964553

[12] Pearson S. Anatomy: Beyond the COVID-19 Pandemic. Acad Med. 2020;95(11):e1. doi: 10.1097/ACM.0000000000003567 32639261 PMC7363372

[13] Tabatabai S. COVID-19 impact and virtual medical education. J Adv Med Educ Prof. 2020;8(3):140–3. doi: 10.30476/jamp.2020.86070.1213 32802908 PMC7395196

[14] Weissmann Y, Useini M, Goldhahn J. COVID-19 as a chance for hybrid teaching concepts. GMS J Med Educ. 2021;38(1):Doc12. doi: 10.3205/zma001408 33659617 PMC7899104

[15] Austin A, Rudolf F, Fernandez J, Ishimine P, Murray M, Suresh P, et al. COVID-19 Educational Innovation: Hybrid In-Person and Virtual Simulation for Emergency Medicine Trainees. AEM Educ Train. 2021:e10593. doi: 10.1002/aet2.10593 33786409 PMC7995095

[16] Remtulla R. The Present and Future Applications of Technology in Adapting Medical Education Amidst the COVID-19 Pandemic. JMIR Med Educ. 2020;6(2):e20190. doi: 10.2196/20190 32634107 PMC7395249

[17] Hayat AA, Keshavarzi MH, Zare S, Bazrafcan L, Rezaee R, Faghihi SA, et al. Challenges and opportunities from the COVID-19 pandemic in medical education: a qualitative study. BMC Med Educ. 2021;21(1):247. doi: 10.1186/s12909-021-02682-z 33926439 PMC8082480

[18] Sharma D, Bhaskar S. Addressing the Covid-19 Burden on Medical Education and Training: The Role of Telemedicine and Tele-Education During and Beyond the Pandemic. Front Public Health. 2020;8:589669. doi: 10.3389/fpubh.2020.589669 33330333 PMC7728659

[19] Owolabi J, Bekele A. Implementation of Innovative Educational Technologies in Teaching of Anatomy and Basic Medical Sciences During the COVID-19 Pandemic in a Developing Country: The COVID-19 Silver Lining? Adv Med Educ Pract. 2021;12:619–25. doi: 10.2147/AMEP.S295239 34135653 PMC8197662

[20] Prabhath S, Dsouza A, Pandey AK, Pandey AK, Prasanna LC. Changing paradigms in anatomy teaching-learning during a pandemic: Modification of curricular delivery based on student perspectives. Journal of Taibah University Medical Sciences. Forthcoming 2021. 10.1016/j.jtumed.2021.10.014 PMC9170788 35722238

[21] Yoo H, Kim D, Lee YM, Rhyu IJ. Adaptations in Anatomy Education during COVID-19. Journal of Korean Medical Science. 2021;36(1):e13. doi: 10.3346/jkms.2021.36.e13 33398947 PMC7781853

[22] Singal A, Bansal A, Chaudhary P, Singh H, Patra A. Anatomy education of medical and dental students during COVID-19 pandemic: a reality check. Surg Radiol Anat. 2021;43(4):515–21. doi: 10.1007/s00276-020-02615-3 33206209 PMC7672260

[23] Ortadeveci A, Ermez MN, Oz S, Ozden H. A survey study on distance anatomy education: challenges unique to anatomy. Surg Radiol Anat. 2022;44(1):41–7. doi: 10.1007/s00276-021-02772-z 34031717 PMC8143068

[24] Zarcone D, Saverino D. Online lessons of human anatomy: Experiences during the COVID-19 pandemic. Clin Anat. 2022;35(1):121–8. doi: 10.1002/ca.23805 34704281 PMC9298225

[25] Kim DH, Shin DH, Hwang YI. Effects of alternate dissection on anatomy learning. Anat Cell Biol. 2019;52(1):69–75. doi: 10.5115/acb.2019.52.1.69 30984454 PMC6449586

[26] McWatt SC. Responding to Covid-19: A thematic analysis of students’ perspectives on modified learning activities during an emergency transition to remote human anatomy education. Anat Sci Educ. 2021;14(6):721–38. doi: 10.1002/ase.2136 34523241 PMC8652611

[27] Potu BK, Atwa H, El-Din WN, Othman M, Sarwani N, Fatima A, et al. Learning anatomy before and during COVID-19 pandemic: Students’ perceptions and exam performance. Morphologie. Forthcoming 2021. 10.1016/j.morpho.2021.07.003 PMC9376010 34384681

[28] Eladl MA, Jarrahi A, Jabbar H, Al Midani O. Use of Anatomy Applications: Prevalence, Student Perception, and Potential Impact on Anatomy Education. The FASEB Journal. 2019;33(S1):604.3–.3.

[29] Golenhofen N, Heindl F, Grab-Kroll C, Messerer DAC, Bockers TM, Bockers A. The Use of a Mobile Learning Tool by Medical Students in Undergraduate Anatomy and its Effects on Assessment Outcomes. Anatomical Sciences Education. 2020;13(1):8–18. doi: 10.1002/ase.1878 30913369

[30] Davis CR, Bates AS, Ellis H, Roberts AM. Human anatomy: let the students tell us how to teach. Anat Sci Educ. 2014;7(4):262–72. doi: 10.1002/ase.1424 24249485

[31] Williams SR T K, Notebaert A, Sinning A. Difficulty of Dissection: Which Anatomical Regions Are Hardest For Medical Students To Dissect? The FASEB Journal. 2018;32:508.12.

