# Supplementary material for: The impact of the modified schedules of anatomy education on students’ performance and satisfaction: Responding to COVID-19 pandemic in South Korea
Source: PLoS One. 2022 Apr 11;17(4):e0266426. doi: 10.1371/journal.pone.0266426 (PMC9000102; doi:10.1371/journal.pone.0266426)
Supplement: S1 Appendix — (DOCX) [file pone.0266426.s001.docx]

S1 Appendix

**A survey on the operation of human anatomy laboratory sessions (the upper and lower limbs, trunk, and head and neck) in 2020 – Google Forms**

Because of COVID-19, this year’s human anatomy laboratory sessions were inevitably conducted in three classes. Face-to-face laboratories are expected to reduce COVID-19 cases or new infectious diseases. To prepare for that time and reflect on this year’s laboratory, I would like to ask for your opinion on the laboratory sessions. I believe the judgment on the upper and lower limbs, trunk, and head and neck will be different. Therefore, I would like to ask the same questions for each session.

**Section 1: Upper and lower limbs session**

1. Overall, I was satisfied with the face-to-face laboratory sessions.

a. Satisfied

b. Neutral

c. Not satisfied

2. I liked three classes with approximately 50 students in each session.

a. Extremely

b. Very

c. Somewhat

d. Slightly

e. Not at all

3. I made good use of the allotted time when I was not doing cadaver dissection.

a. Extremely

b. Very

c. Somewhat

d. Slightly

e. Not at all

4. Complete anatomy application was helpful in cadaver dissection and learning.

a. Extremely

b. Very

c. Somewhat

d. Slightly

e. Not at all

5. I had difficulty learning anatomical structures because I partially performed cadaver dissection.

a. A lot

b. Somewhat

c. A little

6. I had problems taking the practical examination because I partially completed the cadaver dissection.

a. A lot

b. Somewhat

c. A little

**Section 2: Trunk session**

1. Overall, I was satisfied with the face-to-face laboratory sessions.

a. Satisfied

b. Neutral

c. Not satisfied

2. I liked three classes with approximately 50 students in each session.

a. Extremely

b. Very

c. Somewhat

d. Slightly

e. Not at all

3. I made good use of the allotted time when I was not doing cadaver dissection.

a. Extremely

b. Very

c. Somewhat

d. Slightly

e. Not at all

4. Complete anatomy application was helpful in cadaver dissection and learning.

a. Extremely

b. Very

c. Somewhat

d. Slightly

e. Not at all

5. I had difficulty learning anatomical structures because I partially performed cadaver dissection.

a. A lot

b. Somewhat

c. A little

6. I had problems taking the practical examination because I partially completed the cadaver dissection.

a. A lot

b. Somewhat

c. A little

**Section 3: Head and neck session**

1. Overall, I was satisfied with the face-to-face laboratory sessions.

a. Satisfied

b. Neutral

c. Not satisfied

2. I liked three classes with approximately 50 students in each session.

a. Extremely

b. Very

c. Somewhat

d. Slightly

e. Not at all

3. I made good use of the allotted time when I was not doing cadaver dissection.

a. Extremely

b. Very

c. Somewhat

d. Slightly

e. Not at all

4. Complete anatomy application was helpful in cadaver dissection and learning.

a. Extremely

b. Very

c. Somewhat

d. Slightly

e. Not at all

5. I had difficulty learning anatomical structures because I partially performed cadaver dissection.

a. A lot

b. Somewhat

c. A little

6. I had problems taking the practical examination because I partially completed the cadaver dissection.

a. A lot

b. Somewhat

c. A little
